# Subtalar instability: imaging features of subtalar ligaments on 3D isotropic ankle MRI

**DOI:** 10.1186/s12891-017-1841-5

**Published:** 2017-11-21

**Authors:** Tae Hyung Kim, Sung Gyu Moon, Hong-Geun Jung, Na Ra Kim

**Affiliations:** 1 Department of Radiology, Konkuk University Medical Center, Konkuk University School of Medicine, 120-1 Neungdong-ro, Gwangjin-gu, Seoul, 05030 South Korea; 2 Department of Orthopedic Surgery, Konkuk University Medical Center, Konkuk University School of Medicine, Seoul, South Korea

**Keywords:** Instability, Subtalar, Ligaments, Ankle, MRI, 3D

## Abstract

**Background:**

MRI analysis of subtalar ligaments in the tarsal sinus has not been well performed. We retrospectively investigated the appearance of subtalar ligaments using 3D isotropic MRI and compared imaging findings of subtalar ligaments between patients with subtalar instability (STI) and controls.

**Methods:**

Preoperative MRIs of 23 STI patients treated with arthroscopic subtalar reconstruction were compared to MRIs of 23 age- and sex-matched control subjects without STI. Thickness and width of anterior capsular ligament (ACL) and interosseous talocalcaneal ligament (ITCL) as well as thickness of calcaneofibular ligament (CFL) and anterior talofibular ligament (ATFL) were measured. Abnormalities in ACL, ITCL, CFL, ATFL, cervical ligament, and inferior extensor retinaculum were analyzed.

**Results:**

STI patients had significantly smaller ACL thickness and ACL width than controls (ACL thickness: 1.73 mm vs. 2.22 mm, *p* = 0.007; ACL width: 7.21 mm vs. 8.80 mm, *p* = 0.004). ACL thickness of ≤2.1 mm had a sensitivity of 66.7% and a specificity of 66.7% for diagnosis of STI. ACL width of ≤7.9 mm had a sensitivity of 80.0% and a specificity of 76.2% for the diagnosis of STI. However, thickness and width of ITCL, thickness of CFL, or thickness of ATFL was not significantly different between the two groups. Absence or complete tear of ACL was significantly more frequent in STI patients than that in controls (34.8% vs. 8.7%, *p* = 0.035). Complete tear of CFL and ATFL was more common in STI patients than that in controls, although the difference between the two groups was not statistically significant. Abnormalities of ITCL, cervical ligament, or inferior extensor retinaculum were not significantly different between the two groups.

**Conclusions:**

MRI features of thin or narrow ACLs may suggest STI. Absence or complete tear of ACL was significantly more common in STI patients than that in controls.

## Background

Subtalar instability (STI) is a chronic functional talocalcaneal instability characterized by a combination of anterior movement, medialization, and varus tilt of the calcaneus [1, 2]. STI is usually combined with lateral ankle instability (LAI). Incidence of subtalar joint injury has been reported to be as high as 80% in patients with acute lateral ankle sprain. Approximately 10–25% of patients with LAI have STI [3, 4]. Diagnosis of STI is difficult because clinical symptoms of STI are similar to those of LAI. In addition, there is no optimal assessment for STI [1].

Chronic tear and insufficiency of interosseous talocalcaneal ligament (ITCL), cervical ligament (CL), and calcaneofibular ligament (CFL) have been reported as etiologies of STI [5, 6]. However, anatomy and function of subtalar ligaments remain controversial [5]. Some investigators consider ITCL as the most important stabilizer of the subtalar joint. However, inconsistencies occur in morphologies of ITCL. Cadaver studies have shown that there are two distinct ligaments in the tarsal sinus: ITCL and anterior capsular ligament (ACL) [7, 8]. It has been suggested that ITCL and ACL should be considered as two distinct ligaments since they have unique insertion and running patterns.

Despite the association of subtalar ligaments with STI, little attention was paid to the appearance of subtalar ligaments or the ability of MRI to visualize them. MRI analysis of subtalar ligaments in STI patients has not been well performed yet. Therefore, the objective of this study was to retrospectively evaluate the appearance of subtalar ligaments using 3D isotropic MRI and compare imaging findings of subtalar ligaments between STI patients and controls.

## Methods

### Study population

Our Institutional Review Board approved this retrospective study. The requirement for informed consent was waived due to its retrospective nature. A computerized search of medical and radiological records and clinical chart review identified 47 patients with STI who were surgically treated between January 2013 and August 2015. Twenty-three patients (10 females, 13 males) were selected for final analysis based on the following inclusion criteria: (a) clinical diagnosis of STI, surgical confirmation of the diagnosis, and treatment with subtalar reconstruction; (b) arthroscopic surgery performed less than three months after MRI; (c) MRI performed at our institution according to a standardized protocol; (d) no history of ankle surgery; and (e) aged 17 years or older. A total of 24 patients were excluded, including 15 who underwent preoperative MRI at outside institutions, five who did not undergo surgery within three months after MRI, two patients who had prior history of lateral ankle ligament repair, and two patients who were younger than 17 years.

Mean age of patients included in this study was 31.3 years (range, 17 to 57 years). The mean age of female patients was 32.7 years (range, 17 to 57 years). Mean age of the 13 male patients was 30.2 years (range, 18 to 50 years). Mean height, weight, and BMI of STI patients were 168.6 ± 10.5 cm, 71.1 ± 13.4 kg, and 24.9 ± 3.8 kg/m^2^, respectively. Twelve patients were overweight (BMI greater than 25) and two patients were obese (BMI greater than 30). A total of 17 right ankles and 6 left ankles were included. Ten (43.5%) of these 23 ankles also had LAI. All 23 ankles had previous ankle sprain history and preoperative symptomatic recurrent ankle sprain. The mean duration of symptoms was 3.8 years (range, 1 to 11 years). Surgical treatment was performed in patients who did not show symptom improvement despite functional rehabilitation treatment such as peroneal tendon strengthening exercises for ≥3 months.

**Fig. 1:**
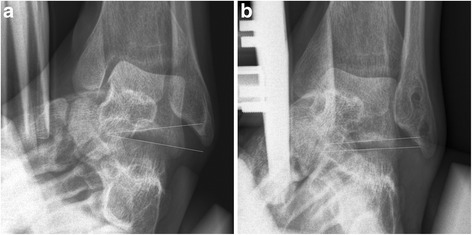
Subtalar tilt angle on Broden’s stress view. Subtalar tilt angle is defined as the angle between lines drawn across the articular surface of the talus and the calcaneus. **a** Preoperative subtalar tilt angle was measured 15 degrees. **b** At 6 months postoperatively, the subtalar tilt angle decreased to 1 degree

Preoperative clinical diagnosis of STI was based on the following diagnostic criteria provided by the senior orthopedic surgeon in our hospital [6]: patients who met at least four of the following five features of preoperative diagnostic criteria: 1) recurrent ankle sprain, 2) sinus tarsi pain and tenderness, 3) hindfoot looseness or giving way, 4) hindfoot instability on physical examination, and 5) radiographic STI on ankle and Broden’s varus stress radiographic views. Ankle and Broden’s varus stress radiographic views were obtained with a Telos SE 2000 stress device (ARD MedizinProdukte GmbH, Marburg, Germany) using 150 Newton of varus stress–force applied at the hindfoot. Positive response on Broden’s varus stress view was defined as an ipsilateral subtalar tilt angle of greater than 10 degrees and a subtalar tilt difference of greater than 5 degrees compared to the contralateral ankle [9] (Fig. 1). Preoperative MRI was performed to determine any additional pathologic condition (such as lateral ankle ligament tear and osteochondral lesion of the talus) that could influence surgical procedure.

For surgical confirmation of STI, the ankle was examined using C-arm stress fluoroscopy under general or spinal anesthesia. In all patients, STI was confirmed by marked tilting of the calcaneus against the talus with lateral widening of the talocalcaneal joint and medial displacement of the calcaneus relative to the talus. Subtalar arthroscopic examination was conducted to evaluate the presence of marked subtalar joint laxity, chronic interosseous ligament tear, synovitis, and other features. Chronic interosseous ligament tear was observed in all patients. An intact ligament was diagnosed when the continuity of the ligament was preserved. Ligament dysfunction caused by chronic tear was defined as definite discontinuity of the ligament and adhesion of adjacent tissue. In 10 cases with both STI and LAI, the Broström procedure was performed in addition to the subtalar reconstruction procedure. Eleven (47.8%) patients had ankle synovitis. Thirteen (56.5%) ankles had subtalar synovitis. Debridement and synovectomy were performed for all patients with synovitis. Combined operations were performed for seven ankles. Two ankles had osteochondral lesion of the talus which was treated by arthroscopic debridement and microfracture. Os subfibulare excision was performed for four ankles. Loose-body removal was performed for one ankle. Lateral sliding calcaneal osteotomy was performed for one ankle with cavovarus deformity.

The control group consisted of 23 subjects who underwent ankle MRI based on a standardized protocol in our institution. They did not show any clinical or arthroscopic sign of STI. Matching criteria for control subjects were: age range, 18–55 years; mean age, 31.9 years; age range of women, 18–55 years; mean age of women, 30.0 years; age range of men, 19–52 years; mean age of men, 32.3 years; sex, 10 women and 13 men. Mean height, weight, and BMI of control subjects were 168.5 ± 8.4 cm, 68.0 ± 16.9 kg, and 23.8 ± 4.8 kg/m^2^, respectively. Nine subjects were overweight (BMI greater than 25) and three subjects were obese (BMI greater than 30). A total of 13 right ankles and 10 left ankles were included. They were diagnosed as acute ankle sprain (*n* = 6), post-traumatic soft tissue impingement (*n* = 4), osteochondral lesion of the talus (n = 4), inflammatory arthritis (n = 4), achilles tendinopathy (*n* = 3), and peroneus tenosynovitis (*n* = 2). Of these 23 subjects, seven underwent ankle and subtalar arthroscopic examinations. They were confirmed to have no STI. For the control group, the mean follow-up period after ankle MRI was 21 months (range, 6–42 months).

### MRI protocol

MR exams were performed using two 3.0-T MRI units with dedicated coils, including a Magnetom Skyra (Siemens Healthcare Diagnostics, Erlangen, Germany) using a sixteen-channel (Siemens Healthcare Diagnostics) ankle coil and a Signa HDxt (GE Healthcare, Milwaukee, WI, USA) with an eight-channel (GE Healthcare) coil. A 3D T2-weighted FSE imaging sequence was used in the sagittal plane without fat suppression. Using Magnetom Skyra, 3D data were acquired with a slice thickness of 0.5 mm using the following imaging parameters: repetition time, 1200 ms; echo time, 155 ms; flip angle, 120°; echo train length, 61; bandwidth, 360 kHz/pixel; field of view, 140 mm; and matrix, 256 × 230. Using Signa HDxt, 3D data acquisition was performed with a slice thickness of 0.4 mm and the following imaging parameters: repetition time, 1250 ms; echo time, 63 ms; flip angle, 90°; echo train length, 34; bandwidth, 195 kHz/pixel; field of view, 140 mm; and matrix, 256 × 224. Subsequently, sagittal images originally acquired from 3D data were reformatted into axial and coronal images with a slice thickness of 0.6 mm without interslice gap. Other 2D imaging sequences including axial and coronal T2-, sagittal T1-, sagittal T2- with fat suppression, and axial, coronal, sagittal T1-weighted images with contrast enhancement were also acquired.

### MRI analysis

#### Quantitative analysis

**Fig. 2:**
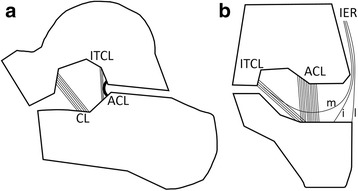
Schematic illustration of ligaments of the sinus tarsi. **a** On the sagittal plane, cervical ligament is located in the anterior aspect of the tarsal sinus, extending from the undersurface of the talar neck to the back of the calcaneus. Interosseous talocalcaneal ligament and anterior capsular ligament are located along the posterior wall of sinus tarsi. **b** On the coronal plane along the posterior wall of sinus tarsi, interosseous talocalcaneal ligament runs obliquely while anterior capsular ligament is vertical in direction. The inferior extensor retinaculum is more laterally positioned with respect to the anterior capsular ligament and interosseous talocalcaneal ligament. CL, cervical ligament; ITCL, interosseous talocalcaneal ligament; ACL, anterior capsular ligament; IER, inferior extensor retinaculum; m, medial root of IER; i, intermediate root of IER; l, lateral root of IER

Schematic illustrations of ligaments in the sinus tarsi are shown in Fig. 2. CL was located in the anterior part of the sinus tarsi, extending from the inferior-lateral aspect of the talar neck to the dorsal surface of the calcaneal neck. ITCL and ACL were located along the posterior wall of the sinus tarsi. On the coronal plane along the posterior wall of the sinus tarsi, ITCL coursed obliquely. However, ACL was vertical like a curtain. This flat thick ligament was defined as thickened segment of the anterior joint capsule of the posterior talocalcaneal facet. ACL originated at the anterior border of the posterior facet of the talus. It ran vertically across the subtalar joint before attaching to the calcaneus [7]. ITCL was located in the anteromedial side to the ACL. It ran obliquely from the talus in the tarsal canal toward the calcaneus in the tarsal sinus [7]. The space between ITCL and ACL was filled with adipose tissue. Therefore, ACL and ITCL could be clearly distinguished from each other.

**Fig. 3:**
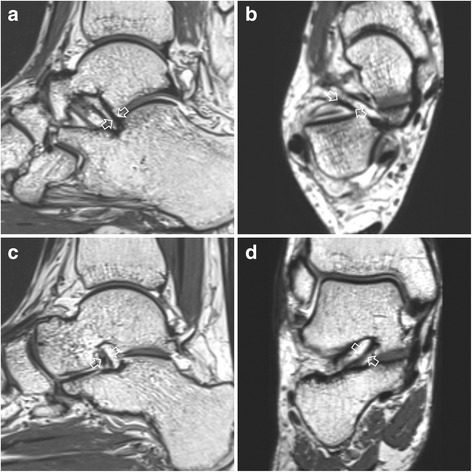
A 33-year-old woman with ankle sprain. Sagittal (**a**) and axial (**b**) isotropic 3D T2-weighted images demonstrating the thickness and width of anterior capsular ligament (open arrows), respectively. Sagittal (**c**) and coronal (**d**) isotropic 3D T2-weighted images demonstrating the thickness and width of interosseous talocalcaneal ligament (open arrows), respectively

Ligament dimensions were measured in the plane that best represented the structure. For ACL, thickness and width were measured on sagittal and axial isotropic 3D T2 weighted images, respectively (Fig. 3). Thickness and width of ITCL were obtained from isotropic 3D T2 weighted images in sagittal and coronal planes, respectively (Fig. 3). Thickness of CFL and ATFL were also measured in axial isotropic 3D T2 weighted image. These measurements were performed at the center of the ligament except for CFL. Thickness of the CFL was measured at the mid-portion between peroneal intersection and calcaneal attachment. Quantitative measurements were obtained thrice by one investigator. Mean values were recorded in millimeters. All measurements were performed using measurement tools included in the PACS computer imaging system.

#### Qualitative analysis

The following qualitative criteria were evaluated and characterized as present or absent: (a) abnormalities of ACL and ITCL characterized by the absence or complete tear of ligaments, (b) abnormalities of CFL and ATFL characterized by complete tear of ligaments, (c) abnormalities of CL characterized by complete tear, (d) abnormalities of inferior extensor retinaculum characterized by partial or complete absence of three roots of inferior extensor retinaculum. Diagnostic criteria for determining complete tear of the ligament included non-visualization of the ligament, discontinuity, and a wavy or curved contour [10]. MRI was evaluated by two musculoskeletal radiologists (with 17 and 5 years of experience, respectively) who were blinded to the diagnosis. Each reader independently evaluated the status of ligaments and subsequently reviewed them to determine the status in consensus. Additionally, edema or obliteration of tarsal sinus fat, and synovial recess extension into tarsal sinus were evaluated in consensus using 2D imaging sequences with or without contrast enhancement.

### Statistical analysis

Continuous data were analyzed with Mann-Whitney test. Receiver operating characteristic (ROC) analysis was used to determine cutoff values of ACL thickness and width for discrimination between the two groups. Sensitivity and specificity were calculated for quantitative criteria and cutoff values of ACL thickness and width. Fisher’s exact test was used to compare qualitative criteria. Interobserver agreement was calculated using kappa statistics based on the following criteria: κ < 0, no agreement; 0 < κ ≤ 0.2, slight agreement; 0.2 < κ ≤ 0.4, fair agreement; 0.4 < κ ≤ 0.6, moderate agreement; 0.6 < κ ≤ 0.8, substantial agreement; 0.8 < κ ≤ 1, almost perfect agreement [11]. Statistical analysis was performed using SPSS for Windows version 21.0 (SPSS, Chicago, IL, USA). A *p* value of less than 0.05 was considered statistically significant.

## Results

Mean BMI was 24.9 ± 3.8 kg/m^2^ for the STI patient group and 23.8 ± 4.8 kg/m^2^ for the control group. There was no significant difference in BMI between STI patient group and the age- and sex-matched control group (*p* = 0.223, Mann-Whitney test).

All patients underwent C-arm stress fluoroscopy under anesthesia. They showed positive STI findings with marked widening of the subtalar joint. Chronic tears in the interosseous ligament were recorded in all cases during subtalar arthroscopy. For 10 cases diagnosed with both LAI and STI, the Broström procedure was also performed in addition to subtalar reconstruction. Semi-tendinous allograft was used to reconstruct anterior and posterior CFL during subtalar reconstruction surgery [6].

### Quantitative analysis of MRI findings

**Fig. 4:**
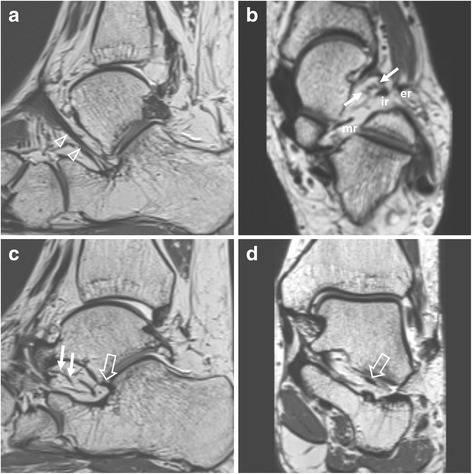
A 27-year-old woman with subtalar instability. Sagittal (**a**) and axial (**b**) isotropic 3D T2-weighted images demonstrating intact inferior extensor retinaculum (arrowheads). er, ir, and mr indicate external, intermediate, and medial root of inferior extensor retinaculum, respectively. Cervical ligament represents a bundle of fan-shaped striated fibers (solid arrows). Sagittal (**c**) and coronal (**d**) isotropic 3D T2-weighted images demonstrating a thin and narrow anterior capsular ligament (open arrows). Solid arrows indicate cervical ligament with thin sheet-like fan-shaped striped fiber bundles

The STI patient group had significantly smaller ACL thickness and width than the control group (thickness: 1.73 ± 0.58 mm vs. 2.22 ± 0.49 mm, *p* = 0.007; width: 7.21 ± 1.69 mm vs. 8.80 ± 1.50 mm, *p* = 0.004) (Fig. 4). Thickness of ITCL, width of ITCL, thickness of ATFL, or thickness of CFL was not significantly different between the two groups (Table 1).

**Table 1:** Quantitative analysis of ligaments between subtalar instability patients and controls

Ligaments		Patient (mm)	Control (mm)	*p*-value
ACL	Thickness	1.73 ± 0.58	2.22 ± 0.49	0.007
	Width	7.21 ± 1.69	8.80 ± 1.50	0.004
ITCL	Thickness	2.07 ± 0.96	2.08 ± 0.78	0.860
	Width	2.75 ± 1.07	2.85 ± 1.13	0.991
CFL	Thickness	2.37 ± 1.17	2.34 ± 0.65	0.538
ATFL	Thickness	3.14 ± 1.11	3.63 ± 1.10	0.178

Note. *P* values were determined using Mann-Whitney test. Data are presented as averaged measurement results ± standard deviation

*ACL* anterior capsular ligament, *ITCL* interosseous talocalcaneal ligament, *CFL* calcaneofibular ligament, *ATFL* anterior talofibular ligament

Based on ROC analysis of ACL dimensions, a cutoff of 2.1 mm for ACL thickness had a sensitivity of 66.7% and a specificity of 66.7% (AUC = 0.765; *p* = 0.007) for STI diagnosis while a cutoff of 7.9 mm for ACL width had a sensitivity of 80.0% and a specificity of 76.2% (AUC = 0.778; *p* = 0.005) to distinguish STI patients from controls.

### Qualitative analysis of MRI findings

**Fig. 5:**
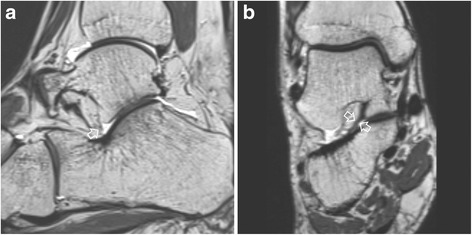
A 25-year-old women with subtalar instability. **a** Sagittal isotropic 3D T2-weighted image demonstrating the absence of anterior capsular ligament (open arrow). **b** On a coronal image along the posterior wall of sinus tarsi, the interosseous talocalcaneal ligament (open arrow) appears to be intact

Other ligament abnormalities besides ACL abnormalities were not significantly different between the two groups (Table 2). In the STI patient group, four cases had no ACL while another four had complete tear of ACL (Fig. 5). In the control group, there were two cases without ACL. The absence or complete tear of ACL was significantly more frequent in STI patients compared to that in controls (34.8% vs. 8.7%, *p* = 0.035). Compared to controls, STI patients had more percentages of complete tear of CFL (17.4% vs. 4.3%, *p* = 0.173) and complete tear of ATFL (17.4% vs. 8.7%, *p* = 0.333), although differences between the two groups were not statistically significant. There was no case of absence or complete tear of ITCL in either group. Based on its shape, ITCL was classified into three categories: band type (*n* = 38, 82.6%), fan type (*n* = 4, 8.7%), and split type (n = 4, 8.7%). There was no significant (*p* = 0.368) difference in the type of ITCL shape between STI and control groups. CL was well visualized on coronal and sagittal planes. It was identified 100% in both groups. CL irregularity and thinning were observed in two cases of the STI patient group. The remaining cases in both groups showed fan or band-shape striated fiber bundles. Partial absence of IER was found in two cases of the STI patient group. One of them showed no intermediate or medial root. The other one showed no medial root. However, the lateral root was visualized in all subjects. In all study subjects except two, the medial root was blended with fibers of the ITCL to form a common insertion.

**Table 2:** Qualitative analysis of ligaments between subtalar instability patients and controls

MRI findings	Patient (*n* = 23)	Control (n = 23)	*p*-value
Absence or complete tear of ACL	8 (34.8%)	2 (8.7%)	0.035
Absence of ITCL	0	0	
Complete tear of CFL	4 (17.4%)	1 (4.3%)	0.173
Complete tear of ATFL	4 (17.4%)	2 (8.7%)	0.333
Irregularity or thinning of CL	2 (8.7%)	0	0.244
Partial absence of IER	2 (8.7%)	0	0.244

Note. *P* values were determined using the Fisher’s exact test. Data are presented as number (percent) of patients

*ACL* anterior capsular ligament, *ITCL* interosseous talocalcaneal ligament, *CFL* calcaneofibular ligament, *ATFL* anterior talofibular ligament, *CL* cervical ligament, *IER* inferior extensor retinaculum

Interobserver agreement between the two readers was considered substantial with kappa values of 0.663 for abnormalities of ACL, 0.726 for ITCL, 0.732 for ATFL, 0.691 for CFL, 0.726 for CL, and 0.646 for IER. Total number of discrepant reads was 18 (six in ACL, three each in ATFL and CFL, and two each in ITCL, CL and IER). All discordantly interpreted cases were re-reviewed to achieve consensus between the two readers. Eleven of them were in favor of reader 1 (four in ACL, one in ITCL, and two each in ATFL, CFL and IER). The rest of them were in favor of reader 2. These two readers were perfectly matched for CL.

Edema of tarsal sinus fat was more frequent in STI patients compared to that in controls (30.4% vs. 21.7%). However, the difference in the percentage of edema of tarsal sinus fat between the two groups was not statistically significant (*p* = 0.369). Synovial recess from the posterior subtalar joint frequently extended into the tarsal sinus, without significant difference between STI patients and controls (47.8% vs. 43.5%, *p* = 0.500). None of all study population demonstrated significant obliteration of tarsal sinus fat.

## Discussion

STI tends to be diagnosed late because it is difficult to distinguish it from LAI on physical examination or stress radiography due to complex joint motion and small changes in laxity [12, 13]. Untreated chronic STI can lead to pain, dysfunction, deformity, and potentially degenerative arthritis. Thus, early diagnosis of STI is needed [2]. In addition to bony structures, subtalar ligaments also play an important role in maintaining the stability of the subtalar joint [2, 14].

Subtalar ligaments are known to consist of CL, ITCL, ACL, and three roots of IER. However, controversy remains regarding which ligament is a more important stabilizer [5, 6]. Some reports have indicated that the CFL is the most important primary stabilizer for the subtalar joint while others have indicated that the ITCL or CL is the most important stabilizer [2, 8, 15–17]. Reported description and nomenclature of ligaments have shown many inconsistencies possibly due to subjective differences in the understanding of the anatomy and variation in shapes. Moreover, some of these ligamentous structures might have been confused with each other due to their adjacent positions. The ITCL has been described with different morphologies, including a V shape, an inverted Y shape, a veil extending across the tarsal canal, an oblique band, and a two-layered structure [7, 8, 14, 18]. A consensus on the description of the ITCL is lacking. Nevertheless, ACL and ITCL should be considered as two distinct ligaments based on their unique insertions and running patterns.

A notable subtalar ligament is the ACL. It was initially called an interosseous ligament. Subsequently, it was called an anterior capsular ligament because it was located along the anterior aspect of the posterior talocalcaneal facet [19, 20]. However, this was not mentioned in many later investigations. Recently, Li SY et al. have designated it a posterior capsular ligament because it is found behind the posterior capsule [8]. The ACL has been described as a thick flat ligament connecting the anterior border of the posterior talocalcaneal facet vertically. It is also identified in the same plane as ITCL [7]. Results from cadaver studies have shown the presence of ACL in 78–95% of specimens [7, 8]. To the best of our knowledge, ACL has not been previously described in radiologic literature.

There are relatively few MRI studies involving STI and subtalar ligaments. Previous cadaver studies [7, 8] and MRI studies [21–23] in asymptomatic models have described normal appearances of subtalar ligaments. According to a pediatric study using 3D isotropic proton density MRI [21], ITCL was striated in appearance in all study population with distinct fascicular bundles. CL most often appeared as a striated fiber bundle. It only occasionally demonstrated homogeneous hypo-intensity. The thickness of the CL ranged from 0.6 to 7 mm. Three roots of the IER were distinguishable in all study populations. Root thickness ranged from 0.5 mm to 2 mm. All tarsal sinus ligaments, i.e. CL, ITCL, and IER were well visualized in 3D isotropic proton density MRI. Each ligament had a unique orientation and dimensions with certain variations. Although there were some differences in dimensions, the results of previous studies were mostly consistent with those of our control group.

In the present study, we evaluated imaging features of subtalar ligaments in STI patents using 3D isotropic T2-weighted MRI. In addition, we compared MRI findings of subtalar ligaments between STI patients and controls. Quantitatively, STI patients had significantly smaller ACL in terms of thickness and width. In the control group, ACL width and thickness were 8.80 mm and 2.22 mm, respectively, similar to previous cadaver-study results (width of 10.1 mm and thickness of 2.4 mm) [8]. However, ACL thickness and width were significantly different between STI patient and control groups. Based on ROC analysis of ACL dimensions, a cutoff of 2.1 mm in thickness had a sensitivity of 66.7% and a specificity of 66.7% while a cutoff of 7.9 mm in width showed a sensitivity of 80.0% and a specificity of 76.2% to distinguish between STI and control. Absence or complete tear of the ACL was significantly more common in the STI patient group compared to that in the control group. In the control group, the prevalence of ACL was 91.3%, consistent with previously reported prevalence range of ACL [7].

ITCL thickness or width showed no significant difference between STI and control groups. According to our results, ITCL thickness and width in the control group were 2.08 mm and 2.85 mm, respectively. ITCL thickness of this study was similar to the thickness reported in previous studies. However, ITCL width of this study was much narrower than previously reported. In a cadaver study, ITCL thicknesses has been reported to be 2.3–3.0 mm with width of 8.5–11.0 mm [7]. In addition, medial roots of IER are known to be blended with fibers of ITCL to form a V-shaped large ligamentous lamina in the tarsal sinus [7]. Likewise, we found that the ITCL was mixed with medial roots of the IER in most cases. The reason that the ITCL width was relatively narrower than previously reported might be due to the fact that only main fiber bundles of ITCL that were clearly visualized on 3D isotropic MRI were measured.

Abnormalities of ITCL, CL, and IER characterized by complete or partial tear were not significantly different between the two groups. In most subjects of both groups, the CL was observed in the shape of a fan or band. Only two STI patients showed irregular or thin CL. In the control group, the CL was best visualized in the coronal plane with 100% rate of detection, similar to the detection rate previously reported in normal pediatric population [21]. Three roots of the IER were distinguished in all subjects except two in the present study. The medial root penetrated the tarsal sinus and blended with fibers of the ITCL to form a common insertion. ITCL, CL, and IER were successfully visualized and characterized in three planes at 100% in the control group, supporting the previous report using 3D proton density MRI [21]. Unlike previous reports, our results suggest that ITCL and CL may not be major stabilizers. Instead, ACL might play a more important role in maintaining the stability of the subtalar joint. ACL can be more important in restraining the posterior talocalcaneal joint due to its course. ACL lies closer to the subtalar joint than CL. It travels more laterally than ITCL. Therefore, it can serve as a central core ligament between the front CL and the rear CFL. In contrast, ITCL is located inside the tarsal sinus. It may not play a major role in restraining varus tilt of the talocalcaneal joint.

Quantitatively, the thickness of CFL or ATFL was not significantly different between the two groups. Complete tears of CFL and ATFL were more frequently observed in STI patients than those in controls, although the difference between the two groups was not statistically significant. In our study, 10 cases in the STI patient group were accompanied by LAI. In the control group, 14 cases had history of lateral ankle sprain. Therefore, the inclusion of lateral ankle sprain might have led to the no significant difference in complete tear of CFL or ATFL between the two groups.

Except CL, other subtalar ligaments including ITCL, three roots of IER, and ACL are located in a small space consisting of the tarsal canal and posterior part of the tarsal sinus. This has led to confusion about ligament anatomy. The function of ACL and ITCL in the tarsal sinus remains unclear due to the lack of anatomical studies. Our results indicate that dimensions of ACL are larger than those of ITCL, especially the width. Dimensions may reflect functional requirements. Furthermore, there was a significant difference in ACL dimensions between the two groups. The cutoff of 2.1 mm in thickness and 7.9 mm in width can facilitate the diagnosis of STI. Until recently, ACL has received little attention in the radiologic field. Further research on functional anatomy and imaging is needed.

Edema of tarsal sinus fat was more common in STI patients. However, there was no significant difference between the two groups. Synovial recess from the posterior subtalar joint often extended into the sinus tarsi in both groups. However, none of our study populations demonstrated significant obliteration of tarsal sinus fat. Edema of tarsal sinus fat can be reversible and may be caused by hemorrhage or inflammation with or without tears of the associated ligaments. Edema or obliteration of tarsal sinus fat are known to imply sinus tarsi syndrome, but sinus tarsi syndrome do not mean STI because it can be associated with other ankle diseases as well as STI. Sinus tarsi syndrome usually occurs after inversion injury and is often associated with tear of the lateral collateral ligament [24, 25]. Unlike fat suppression images, 3D isotropic T2-weighted images without fat suppression allowed us to distinguish the ligament boundaries and measure the dimensions because the ligaments had a unique direction and they were more clearly distinguished from the surrounding fat edema. 3D isotropic images provided the additional advantage of anatomical detail by thin section and multiplanar reformation capability, making it easy to track the course and integrity of small structures such as subtalar ligaments.

Our study has several limitations. First, the correlation between clinical and imaging outcomes was not fully evaluated due to the small sample size. In addition, it might be difficult to distinguish between pathologic ligaments and anatomic variations. Anatomic variation is beyond the scope of this study because it needs a large-scale study using normal population. This study focused on STI patients with symptoms rather than asymptomatic ankles, unlike most studies. The aim of this study was to compare STI patients and controls by focusing on subtalar ligaments to find unusual findings that might lead to STI. Second, the patient group consisted of STI patients regardless of LAI combination. More specific results can be obtained by selecting patients with LAI without STI as controls. Further research is needed to address this issue. Third, this study focused on ligamentous structures of the tarsal sinus and lateral ankle. However, other factors such as bony structure might also play a role in maintaining joint stability. Nevertheless, we tried to assess all candidate subtalar ligaments including ACL. Fourth, chronicity of ligament tear that might affect MRI findings was not evaluated in this study. Even though ligaments might appear intact, they could be thinned or thickened by prior partial tears without being detected. This can add bias to the diameters in the current study. Last, due to the retrospective nature of the study, clinical information and radiological evaluation might have introduced a bias.

## Conclusions

Thin or narrow ACL MRI findings might suggest STI. Absence or complete tear of ACL was significantly more common in STI patients than that in controls. Since STI is usually combined with LAI, complete tears of CFL and ATFL are common in STI.

## Abbreviations

ACL: Anterior capsular ligament
ATFL: Anterior talofibular ligament
CFL: Calcaneofibular ligament
CL: Cervical ligament
ITCL: Interosseous talocalcaneal ligament
LAI: Lateral ankle instability
STI: Subtalar instability
